# Supplementary material for: Pathoadapative Genomic Determinants of Staphylococcus aureus Community Skin Infections and Nasal Colonization
Source: Microorganisms. 2025 Aug 29;13(9):2023. doi: 10.3390/microorganisms13092023 (PMC12471753; doi:10.3390/microorganisms13092023)
Supplement: Supplementary file 1 [file microorganisms-13-02023-s001.zip › Supplementary Information.pdf]

Pathoadapative Genomic Determinants of *Staphylococcus aureus* Community Skin Infections and Nasal Colonization

Cody A. Black[1,2], Wonhee So[5], Raymond Benavides[1,2], Julianne A Mercer[1,2], Steven S. Dallas[2-4], James Shurko[1,2], Sarah M. Bandy[1,2], Benjamin A. Encino[1,2], Justina Lipscomb[1,2], Christopher R. Frei[1,2,4], Grace C. Lee[1,2,6*]

[1] College of Pharmacy, The University of Texas at Austin, Austin, TX 78712, USA
[2] Joe R. and Teresa Lozano Long School of Medicine, The University of Texas Health at San Antonio, San Antonio, TX 78229, USA
[3] Department of Pathology and Laboratory Medicine, The University of Texas Health at San Antonio, San Antonio, TX 78229, USA
[4] University Health System, 4502 Medical Drive, San Antonio, TX 78229, USA
[5] College of Pharmacy, Western University of Health Sciences, Pomona, CA 91766, USA
[6] Veterans Administration Research Center for AIDS and HIV-1 Infection and Center for Personalized Medicine, South Texas Veterans Health Care System, San Antonio, TX 78229, USA

*Correspondence: Grace C. Lee; leeg3@uthscsa.edu

This file contains additional tables and figures supporting the findings presented in the main manuscript.

Table of Contents

Supplementary Table S1. Clinical and *Staphylococcus aureus* Characteristics

Supplementary Table S2. Clonal complex, plasmid and antimicrobial resistance characteristics

Figure S1. Volcano plot of all test unitigs

Figure S2. QQ-plot of unitigs test unitigs

Supplementary Table S3. Reference genomes

Figure S3. Heatmap of Non-synonymous Nucleotide Variants (NSNV) of 157 *S. aureus* compared NCTC 8325

Figure S4. Treemap of gene ontological biological process (GO-BP) clusters of NSNV genes

Figure S5. Treemap of KEGG clusters of NSNV genes

Supplemental Table S4A. Top 25 NSNV Genes in CC8

Supplemental Table S4B. Top 25 NSNV Genes in CC8 Terms/Pathways

Figure S6. Bar plot of the normalized distribution of the viral taxa identified between Staphylococcus aureus SSTI and nasal colonization phenotypes

Figure S7: Volcano plot of annotated phage gene products between *Staphylococcus aureus* SSTI and nasal colonization phenotypes

Supplemental Table S5. Annotated phage gene products between *Staphylococcus aureus* SSTI and nasal colonization phenotypes

Figure S8. phiIPLA35 gene product association with SSTI vs. nasal colonization Staphylococcus aureus.

Figure S9. Bootstrapped mean differences in wound size for each phiIPLA35 gene product.

Figure S10. Cobalt Constraint-based Multiple Alignment of Gp5

Figure S11. Volcano plot of whole genome copy number variants normalized to multi-locus serotype genes between SSTI and nasal colonization Staphylococcus aureus.

Supplemental Table S6. PUS Score Distribution

Figure S12. Histogram of PUS scores vs. frequency of 157 Patients with *Staphylococcus aureus* isolates collected from purulent cellulitis and nasal colonization

Figure S13. QQ-plot of unitigs associated with PUS score.

Figure S14. Volcano plot of all *Staphylococcus aureus* unitigs.

Supplemental Table S7. Annotation of Pyseer Identified *Staphylococcus aureus* Genes Associated with Increased PUS Score

Supplemental Table S8. CC, *spa*, SCC*mec*, SSTI and wound size

Supplemental Text S1. Metagenomic results

Supplementary Table S1. Clinical and *Staphylococcus aureus* Characteristics			
	**Nasal Colonization (n = 31)**	**SSTI (n = 126)**	***P***
**Clonal Complex, *n* (%)**			**<0.001**
CC5	7 (23)	2 (2)	
CC8	3 (10)	85 (68)	
CC30	7 (23)	0	
CC45	5 (16)	2 (2)	
Other CC	9 (29)	37 (29)	
**Methicillin Phenotype, *n* (%)**			
MSSA	27 (87)	47 (37)	**<0.001**
MRSA	4 (13)	79 (63)	
**Patient characteristics, *n* (%)**			
Male (%)	17 (55)	62 (49)	0.72
Female (%)	14 (45)	63 (50)	0.78
Black (%)	2 (7)	7 (6)	1.0
White (%)	28 (90)	113 (90)	1.0
Hispanic (%)	27 (87)	93 (74)	0.19
Highest Temperature F° (mean (SD))	98.60 (0)	98.11 (1)	**0.007**
Weight (kg) (mean (SD))	92 (21)	89 (26)	0.52
Age (years) (mean (SD))	42 (12)	42 (12)	0.83
BMI (mean (SD))	34 (7)	28 (14)	**0.015**
T2DM (%)	10 (32)	32 (25)	0.59
Healthcare provider (%)	4 (13)	1 (1)	**0.004**
Prior antibiotics (%)	5 (16)	18 (14)	1.0
Prior MRSA	0	5 (4)	0.6
**Wound Characteristics, , *n* (%)**			
Wound size cm (mean (SD))	5.3 (3.3)		
Duration days (mean (SD))	6.80 (7.13)		
Erythema cm (mean (SD))	0.71 (0.5)		
Incision/Drainage (IND %)	63 (50)		
**Wound Location, *n* (%)**			
Head/neck	12 (10)		
Trunk	30 (24)		
Upper Extremity	13 (10)		
Lower Extremity	39 (31)		
Groin	20 (16)		
Axilla	15 (12)		

MSSA: Methicillin susceptible *Staphylococcus aureus*; **MRSA**: Methicillin resistant *Staphylococcus aureus*; BMI: Body mass index; **CC**: clonal complex; **SSTI**: skin and soft tissue infection; **T2DM**: Type 2 diabetes mellitus

Supplementary Table S2. Clonal Complex, Plasmid and Antimicrobial Resistance Characteristics of *Staphylococcus aureus*

Data Type	Description (Count)
**Clonal Complex**	CC8 (88), Novel CC (25), CC5 (9), CC45 (7), CC30 (7), CC188 (4), CC15 (3), CC4236 (2), CC6370 (2), CC59 (2), CC12 (1), CC25 (1), CC7776 (1), CC20 (1), CC72 (1), CC121 (1), CC1159 (1), CC6 (1)
**Plasmid**	*rep16* (123), *rep7c* (115), *rep19* (98), *rep21* (80), *rep5a* (26), *rep20* (15), *rep24a* (9), *rep10* (7), *rep15* (5), *rep13* (5), *rep7a* (2), *rep10b* (2)
**Resistance**	*blaZ* (138), *mecA* (85), *mph*(C) (85), *msr*(A) (85), *aph(3')-III* (81), *ant(6)-Ia* (79), *erm(C)* (7), *erm(A)* (4), ant(9)-Ia (4), aadD (2), tet(K) (2), dfrG (1), *aac(6')-aph(2'')* (1), *bla*$_{OXA-421}$ (1), *bleO* (1)
SCCmec	SCC_IVa (81), SCC_IIb (1), SCC_IVh (1)
***spa* type**	t008 (36), t622 (21), unknown-spa (17), t723 (9), t111 (5), t334 (4), t189 (4), t024 (3), t17749 (2), t4407 (2), t7477 (2), t002 (2), t338 (2), t160 (2), t304 (2), t351 (1), t14027 (1), t2104 (1), t574 (1), t5649 (1), t19002 (1), t9325 (1), t118 (1), t1911 (1), t1578 (1), t9121 (1), t2427 (1), t2229 (1), t4359 (1), t2743 (1), t688 (1), t287 (1), t2306 (1), t277 (1), t129 (1), t1877 (1), t918 (1), t5271 (1), t6238 (1), t091 (1), t132 (1), t094 (1), t15459 (1), t147 (1), t059 (1), t1259 (1), t4229 (1), t6465 (1), t9829 (1), t1987 (1), t316 (1), t275 (1), t267 (1), t871 (1), t282 (1), t1303 (1), t084 (1), t062 (1), t216 (1)
**CC**: clonal complex	

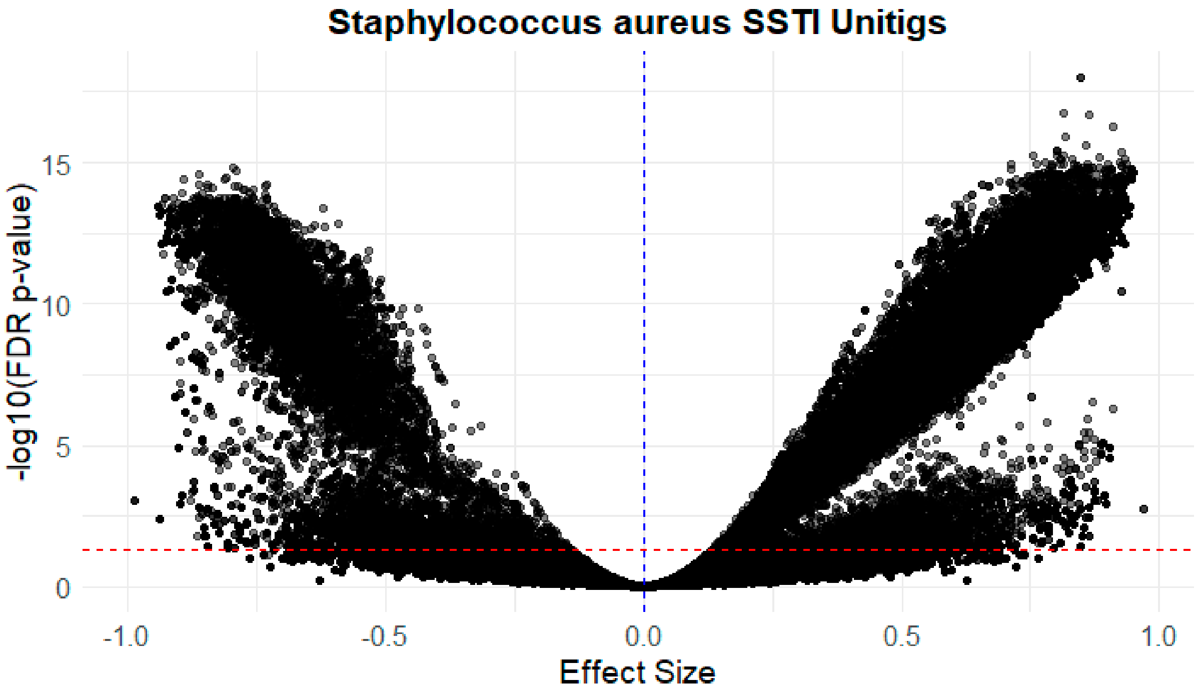

Figure S1. Volcano plot of all test unitigs (n = 303,420). Abbreviations: skin and soft tissue infection (SSTI). Positive effect size corresponds to SSTI associated unitigs, negative effect size corresponds to nasal colonization unitigs.

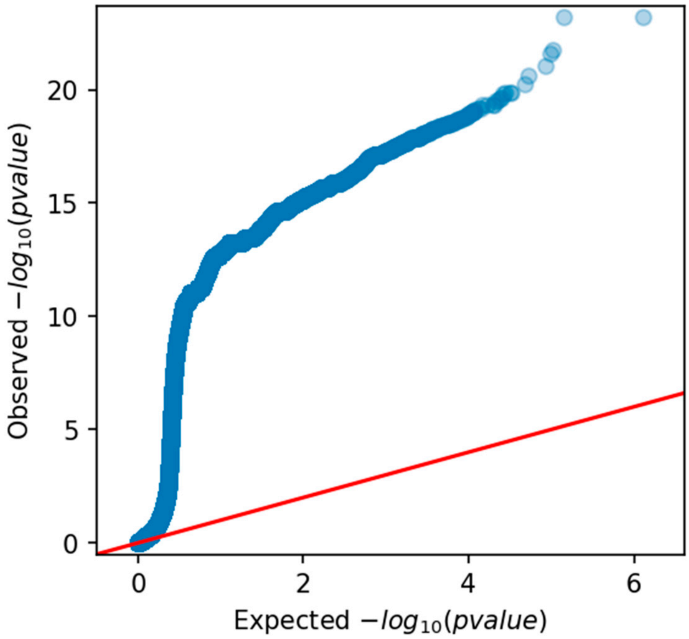

Figure S2. QQ-plot of unitigs test unitigs (n = 303,420).

**Supplementary Table S3.** Reference genomes	
Reference	Accession
VC40	NC_016912.1
04_02981	NC_017340.1
08BA02176	NC_018608.1
6850	NC_022222.1
8325	NC_007795.1
11819_97	NC_017351.1
71193	NC_017673.1
COL	NC_002951.2
ECT_R_2	NC_017343.1
ED98	NC_013450.1
ED133	NC_017337.1
HO_5096_0412	NC_017763.1
JH9	NC_009487.1
JKD6008	NC_017341.1
JKD6159	NC_017338.2
LGA251	NC_017349.1
M013	NC_016928.2
MRSA252	NC_002952.2
MSHR1132	NC_016941.1
MSSA476	NC_002953.3
Mu3	NC_009782.1
Mu50	NC_002758.2
MW2	NC_003923.1
N315	NC_002745.2
Newman	NC_009641.1
RF122	NC_007622.1
ST398	NC_017333.1
T0131	NC_017347.1
TCH60	NC_017342.1
TW20	NC_017331.1
USA300_FPR3757	NC_007793.1
USA300_TCH1516	NC_010079.1

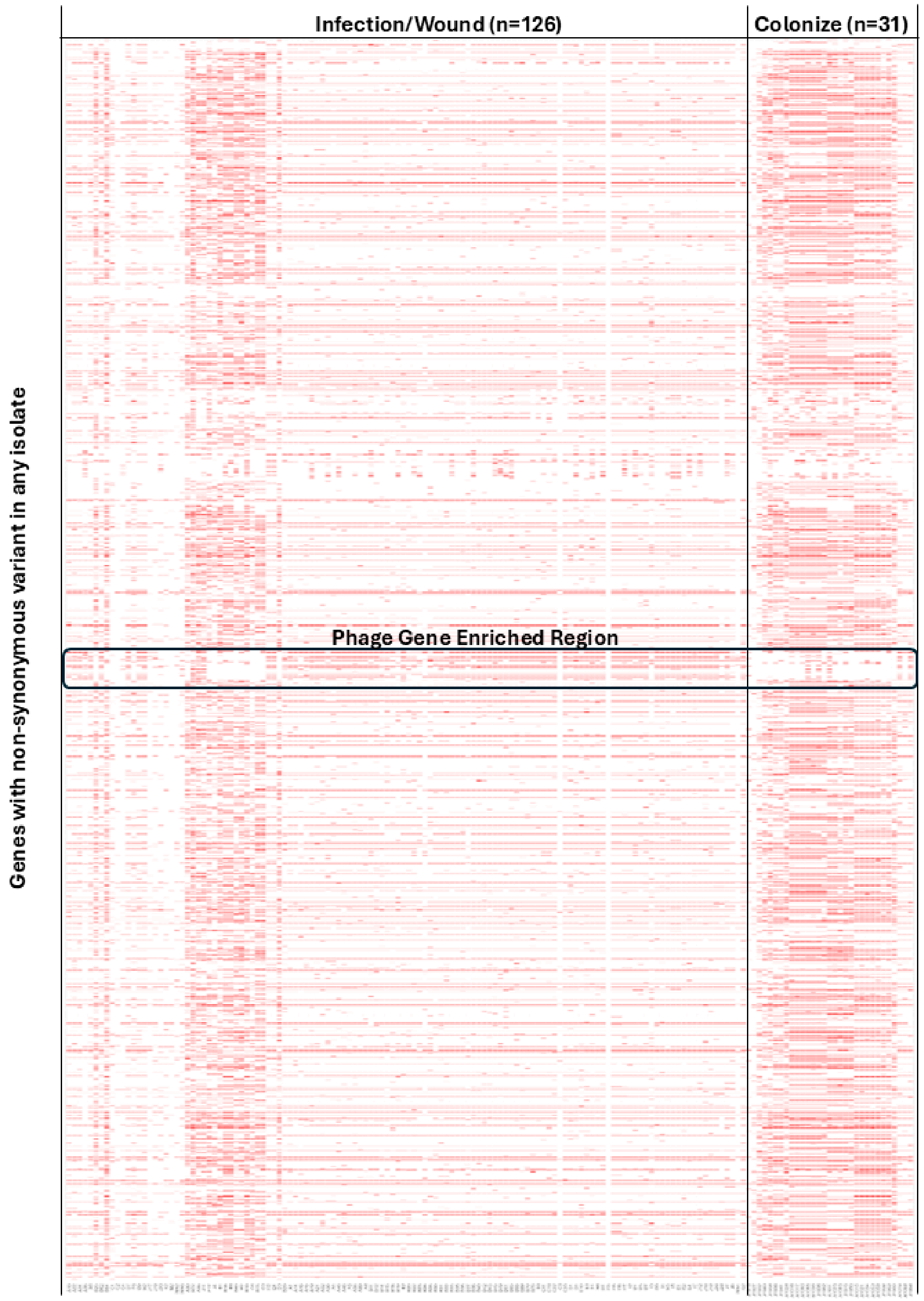

Figure S3. Heatmap of Non-synonymous Nucleotide Variants (NSNV) of 157 *S. aureus* compared NCTC 8325. Gene (y-axis) and sample (x-axis; left-to-right represents cellulitis to colonization source, and MLST). Red cells represent genes with NSNV compared to reference. White cells represent genes which sequence match reference.

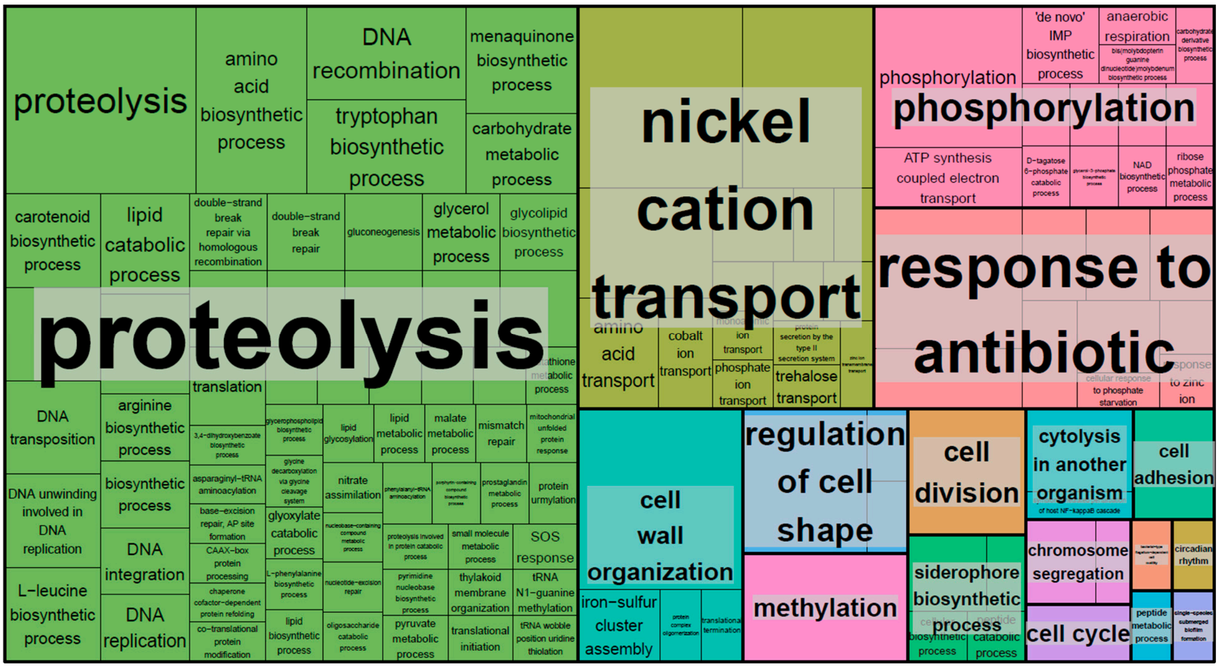

Figure S4. Treemap of gene ontological biological process clusters of non-synonymous nucleotide variants (NSNV) genes with significantly different variant proportions in cellulitis (n=126) or colonize (n=31) phenotypes. Reference genome used: NCTC 8325. The representative/global term for each map area are shown as large overlaid labels.

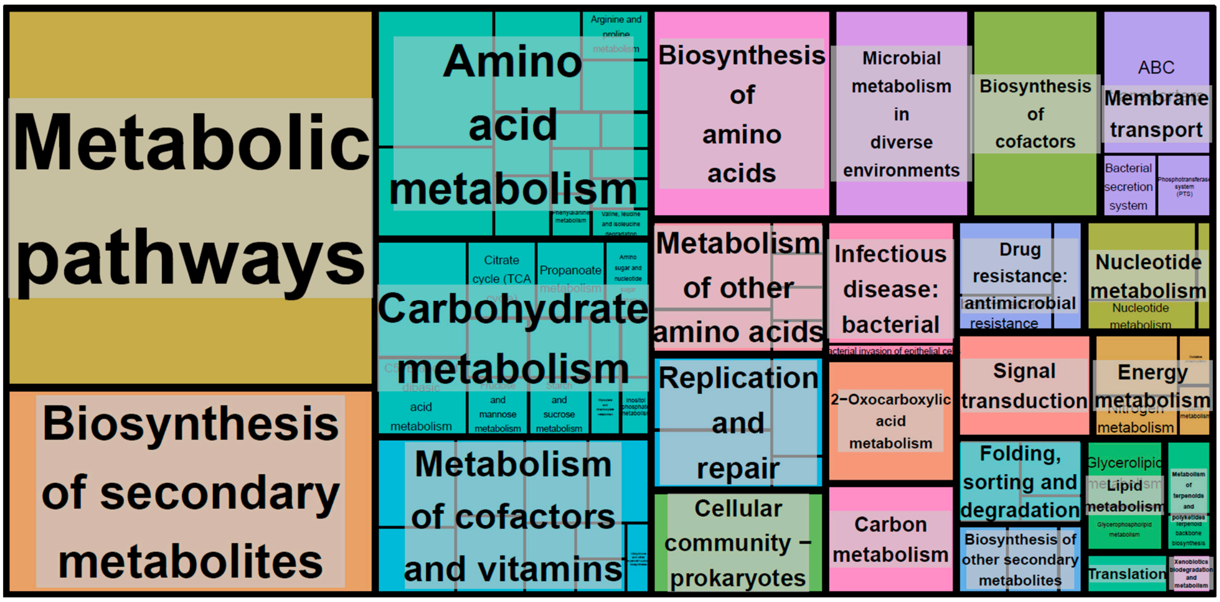

Figure S5. Treemap of KEGG pathways of non-synonymous nucleotide variants (NSNV) genes with significantly different variant proportions in cellulitis (n=126) or colonize (n=31) groups. Reference genome used: NCTC 8325. The representative/global pathways for each map area are shown as large overlaid labels.

Supplemental Table S4A. Top 25 NSNV Genes in CC8

Locus Tag	Product	% Diff.
SAOUHSC_01901	Transaldolase	56
SAOUHSC_02866	SSD domain-containing protein	56
SAOUHSC_02076	Phi PVL orf 38-like protein	38
SAOUHSC_00789	Probable cell division protein WhiA	36
SAOUHSC_01025	Uncharacterized protein	34
SAOUHSC_01582	Bacteriophage integrase	29
SAOUHSC_00654	Ferrichrome ABC transporter (Permease), putative	28
SAOUHSC_01546	Conserved hypothetical phage protein	27
SAOUHSC_01873	LPXTG-anchored repetitive surface protein SasC	27
SAOUHSC_02633	Bcr/CflA family efflux transporter	27
SAOUHSC_02021	Phi ETA orf 63-like protein	27
SAOUHSC_01570	PVL orf 37-like protein	26
SAOUHSC_02205	Conserved hypothetical phage protein	25
SAOUHSC_01528	Bacteriophage L54a	25
SAOUHSC_02048	Phage portal protein, SPP1 family	24
SAOUHSC_01352	DNA topoisomerase 4 subunit A	24
SAOUHSC_01520	SLT orf 488-like protein	23
SAOUHSC_01544	Hypothetical phage protein	23
SAOUHSC_02023	Bifunctional autolysin	23
SAOUHSC_02203	Conserved hypothetical phage protein	23
SAOUHSC_01525	lysostaphin	21
SAOUHSC_01565	Phage-related protein	21
SAOUHSC_02029	Phi ETA orf 56-like protein	21
SAOUHSC_02075	Conserved hypothetical phage protein	20
SAOUHSC_01560	Conserved hypothetical phage protein	19

Reference: NCTC 8325.

Supplemental Table S4B. Top 25 NSNV Genes in CC8 Terms/Pathways

Pathway/Term
Membrane (GO:0016020)
Metabolic pathways (KEGG: sao01100)
DNA Binding (GO:0003677)
Secreted (KW-0964)
Extracellular Region (GO:0005576)
Cell Wall Organization (GO:0071555)
Peptidoglycan Biosynthetic Process (GO:0009252)
Regulation of Cell Shape (GO:0008360)

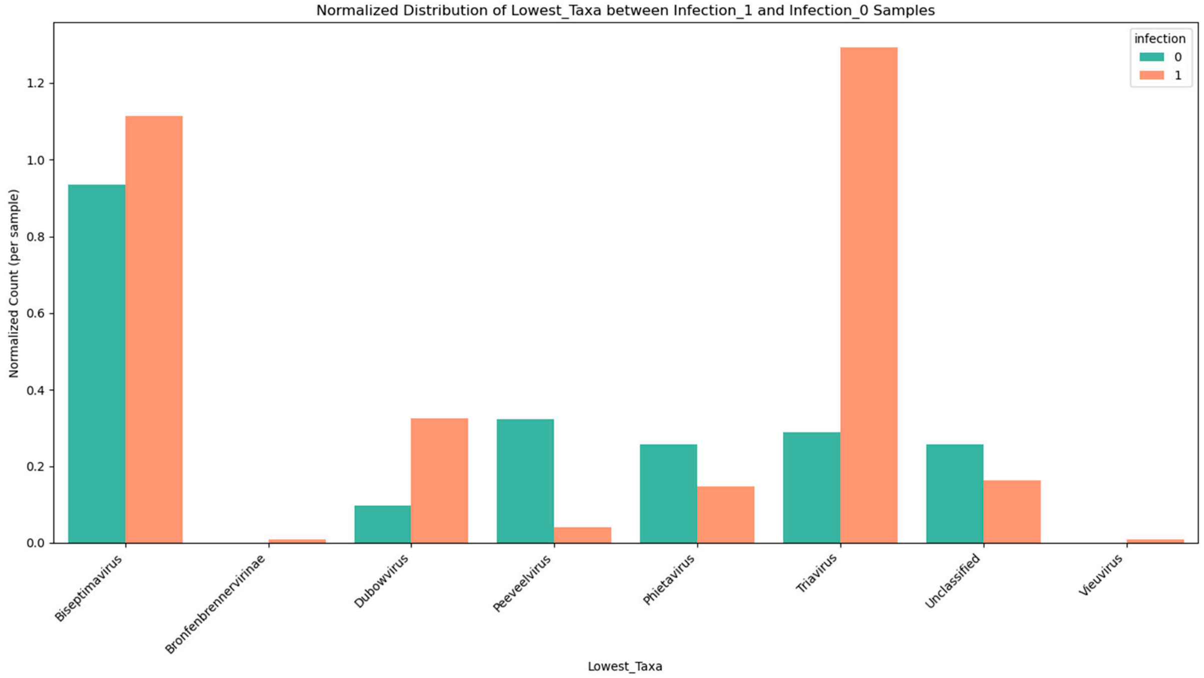

Figure S6. Bar plot of the normalized distribution of the viral taxa identified between *Staphylococcus aureus* SSTI and nasal colonization phenotypes. Counts for each taxa were normalized to the respective sample sizes of SSTI and colonization groups, providing a comparative measure of taxa prevalence per sample. This normalization accounts for variations in group sizes, ensuring that the representation of each taxa reflects its relative abundance within each infection category.

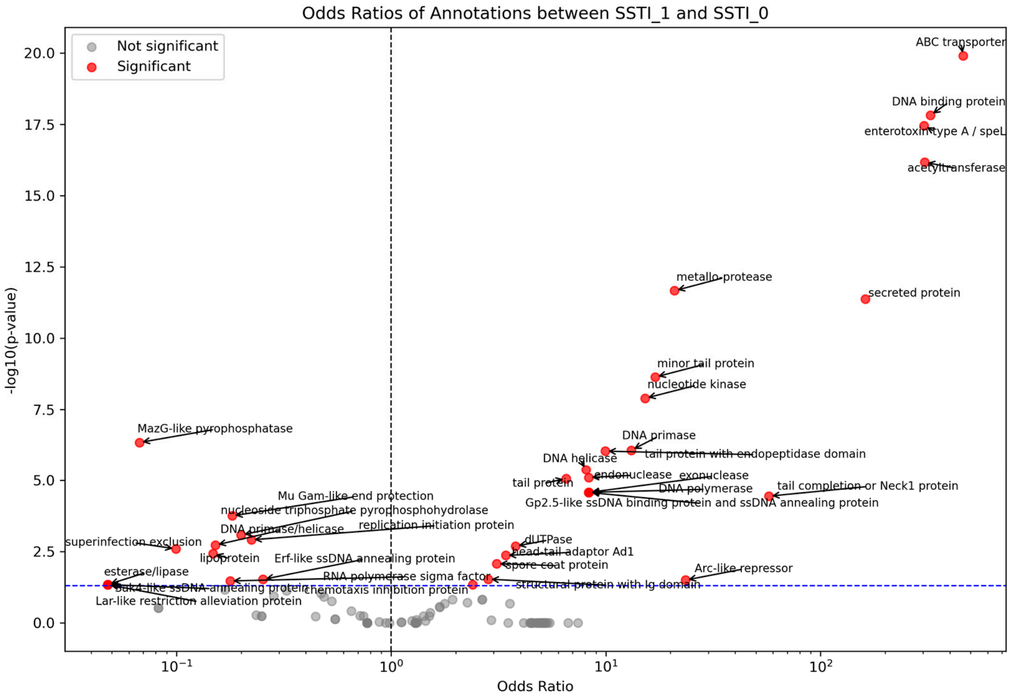

Figure S7: Volcano plot of annotated phage gene products between *Staphylococcus aureus* SSTI and nasal colonization phenotypes. The x-axis represents the odds ratio on a logarithmic scale, while the y-axis represents the -log10(p-value). Points in red indicate annotations with statistically significant differences ($p < 0.05$) between SSTI_1 and SSTI_0 after continuity correction was applied to handle zero counts. The dashed blue line indicates the significance threshold ($p = 0.05$), and the dashed black line represents an odds ratio of 1, indicating no difference between groups. Labels for significant annotations have been adjusted to minimize overlap for better visualization.

**Supplemental Table S5**. Annotated phage gene products between *Staphylococcus aureus* SSTI and nasal colonization phenotypes				
Phage gene annotation	SSTI (n=123)	Colonize (n=31)	P	OR
ABC transporter	1.04878	0.354839	1.24E-20	461.6957
DNA binding protein	1.235772	0.483871	1.52E-18	324.6774
enterotoxin type A / speL	1.308943	0.516129	3.54E-18	303.4242
acetyltransferase	1.02439	0.451613	6.76E-17	305.3448
metallo-protease	0.886179	0.258065	2.16E-12	20.8783
secreted protein	1.341463	0.677419	4.20E-12	161.6512
minor tail protein	0.95122	0.516129	2.31E-09	16.98135
nucleotide kinase	0.715447	0.129032	1.28E-08	15.23474
MazG-like pyrophosphatase	0.03252	0.354839	4.56E-07	0.067128
DNA primase	0.617886	0.096774	8.52E-07	13.11429
tail protein with endopeptidase domain	0.674797	0.16129	9.13E-07	9.933782
DNA helicase	0.674797	0.193548	4.23E-06	8.088319
endonuclease	0.634146	0.16129	7.94E-06	8.312687
tail protein	0.829268	0.419355	8.68E-06	6.533161
DNA polymerase	0.577236	0.129032	2.66E-05	8.322751
Gp2.5-like ssDNA binding protein and ssDNA annealing protein	0.577236	0.129032	2.66E-05	8.322751
exonuclease	0.577236	0.129032	2.66E-05	8.322751
tail completion or Neck1 protein	1.121951	0.83871	3.60E-05	57.49057
Mu Gam-like end protection	0.113821	0.419355	0.000177	0.181465
nucleoside triphosphate pyrophosphohydrolase	0.097561	0.354839	0.000839	0.199844
replication initiation protein	0.121951	0.387097	0.001227	0.222857
DNA primase/helicase	0.04065	0.225806	0.001879	0.151617
dUTPase	0.650407	0.322581	0.002046	3.789272
superinfection exclusion	0.01626	0.16129	0.002532	0.09914
lipoprotein	0.03252	0.193548	0.003739	0.147731
head-tail adaptor Ad1	0.764228	0.483871	0.004313	3.41006
spore coat protein	0.666667	0.387097	0.008391	3.101205
structural protein with Ig domain	0.837398	0.645161	0.029392	2.832243
Erf-like ssDNA annealing protein	0.056911	0.193548	0.029996	0.252559
Arc-like repressor	1.121951	0.935484	0.031674	23.47458
RNA polymerase sigma factor	0.02439	0.129032	0.03396	0.177501
chemotaxis inhibition protein	0.666667	0.451613	0.045238	2.399252
Lar-like restriction alleviation protein	0	0.064516	0.045603	0.047773
Sak4-like ssDNA annealing protein	0	0.064516	0.045603	0.047773
esterase/lipase	0	0.064516	0.045603	0.047773

To further examine the phages present in both groups, we created a BLAST database with our collection of 157 isolates and screened for ≥ 90% amino acid alignment to known phage proteins using the Phastest virus database. Phage protein annotations which were present in the cellulitis isolates ≥ 25% than the colonizing isolates were further analyzed. This revealed numerous proteins enriched in the cellulitis isolates related to the *Staphylococcus* phage phiIPLA35 [Triavirus]. Proteins Gp5 (metallo-protease)c, Gp41 (major capsid protein), and Gp11 (*IS*630 family transposase) represented the top three phiIPLA35 proteins associated with cellulitis infection.

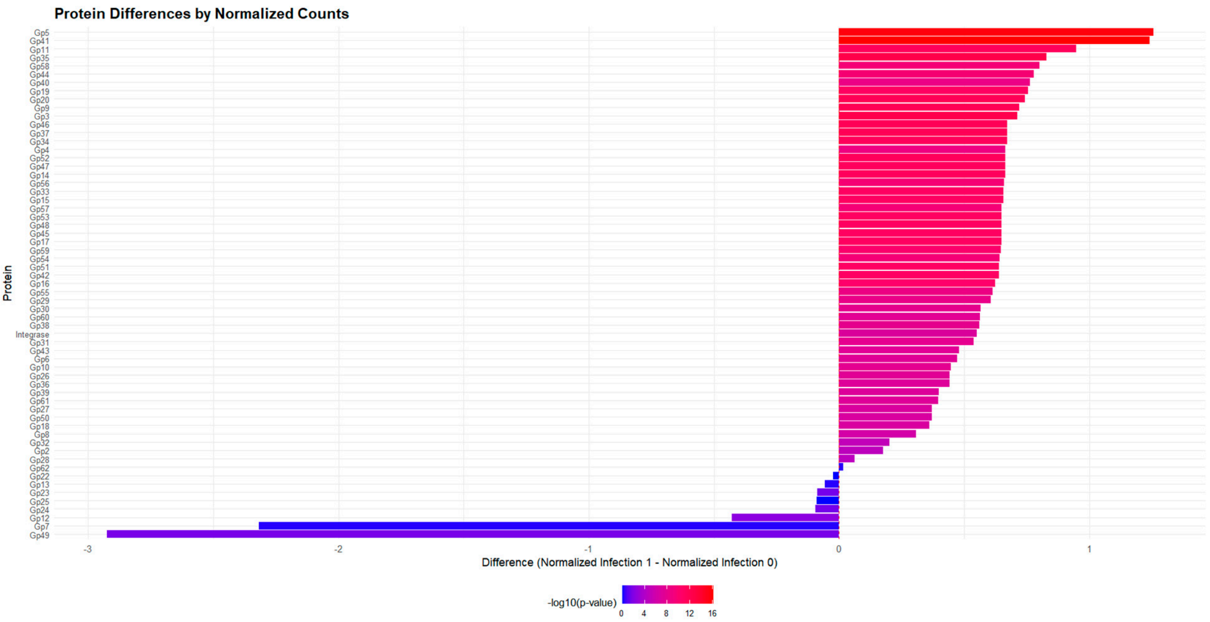

Figure S8. phiIPLA35 gene product association with SSTI vs. nasal colonization *Staphylococcus aureus*. This horizontal bar plot displays the normalized proportion (N = 157; Infection/cellulitis = 126; Colonize = 31) of protein presence for samples containing each specific protein, calculated using the Wilcoxon rank-sum test to compare the differences between samples with the protein present versus absent, with a focus on the infection status (1 or 0). The bars represent the normalized proportion associated with cellulitis infection, while the color gradient reflects the statistical significance of the association, represented as −log10 of the p-value. A more intense red color indicates a stronger statistical association between the protein and infection status. The vertical dashed line at x = 0 serves as a reference point, with proteins to the right showing a positive association with increased wound size in the infection-positive group. This analysis helps identify proteins potentially linked to infection, highlighting those that may contribute to differences in wound size.

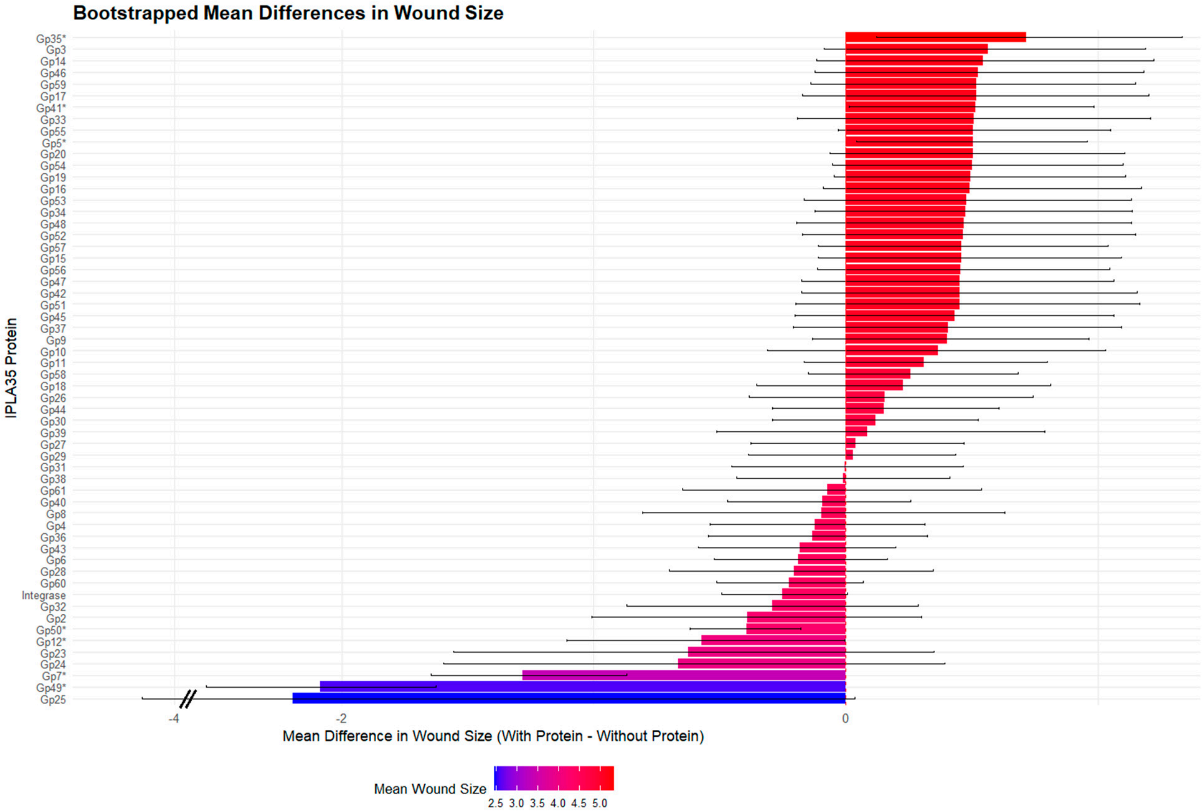

Figure S9. Bootstrapped mean differences in wound size for each phiIPLA35 gene product. The plot displays the mean difference in wound size between samples with and without each protein, estimated using bootstrapping. Each horizontal bar represents the bootstrapped mean difference, with the length of the bar indicating the magnitude of the difference. Error bars show the 95% confidence intervals for the bootstrapped mean differences, providing a range of plausible values. The color gradient of the bars represents the average wound size when the protein is present, with blue indicating smaller mean wound sizes and red indicating larger mean wound sizes. The vertical dashed line at x = 0 marks the point of no difference in wound size. Proteins with error bars not crossing this line are associated with statistically significant differences in wound size. This analysis helps identify proteins that are potentially linked to variations in wound size among samples.

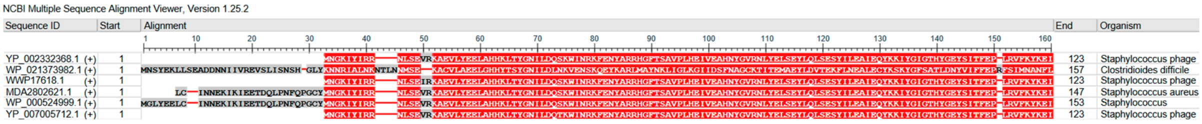

Figure S10. Cobalt Constraint-based Multiple Alignment of Gp5 (YP_002332368.1) with six similar proteins (RID B5J8GXSF212), including a *Clostridoides difficile* ImmA/IrrE family metallo-endopeptidase and other entries for *Staphylococcus aureus* phage toxins.

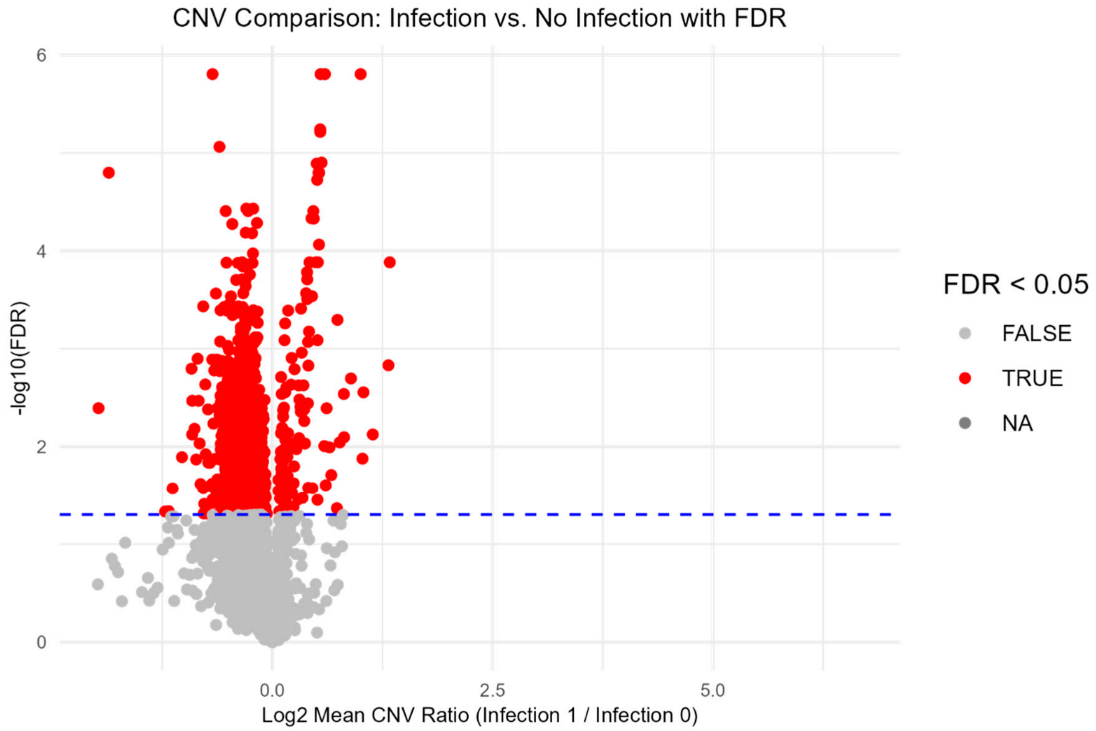

Figure S11. Volcano plot of whole genome copy number variants (CNV) normalized to multi-locus serotype genes between SSTI and nasal colonization *Staphylococcus aureus*. Log10-transformed False Discovery Rate (vertical-axis) and Log2 CNV ratio of SSTI/Colonize (horizontal axis).

The PUS composite score is interpreted using specific thresholds: scores of 5 or higher indicate 'severe,' scores between 3 and 4.9 are 'moderate,' and scores between 1 and 2.9 are 'mild.' A score of 0 indicates a 'colonize' status, signifying no significant clinical findings. Of the 157, this resulted in 17 (11%) mild, 60 (38%) moderate, 49 (31%) severe, and 31 (20%) ‘colonize’ PUS interpretations (**Supplemental Table 6**).

Supplemental Table S6. PUS Score Distribution

PUS Score	Patient (n)	Percent
colonize	31	20
mild	17	11
moderate	60	38
severe	49	31
total	157	100

Abbreviations: **PUS**: Purulence Ulcer Skin score.

Bacterial GWAS was used to associate *S. aureus* unitigs with PUS composite scores. The distribution of PUS composite scores across the 157 patients has a mean of approximately 2.98, with a standard deviation of 2.97. The minimum score is 0, the maximum score is 15, and the majority of the scores fall between 1 and 4, with 50% of the data having a score of 2 or lower. PUS score thresholds were defined as ‘Colonize’: 0; ‘Mild’: >0-2; ‘Moderate’: 2-5; ‘Severe’: ≥5. (**Figure S12**).

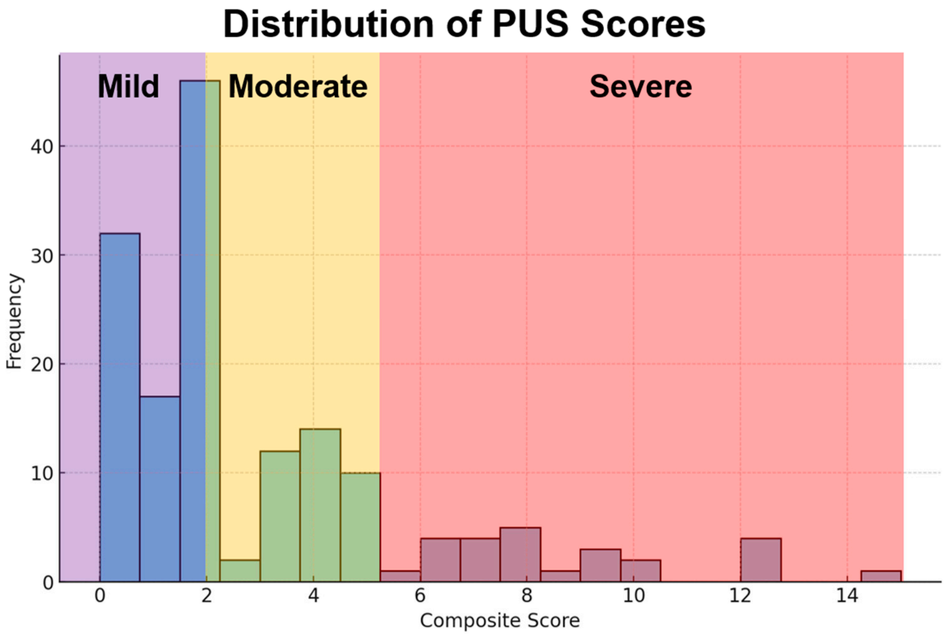

Figure S12. Histogram of PUS scores vs. frequency of 157 Patients with *Staphylococcus aureus* isolates collected from purulent cellulitis and nasal colonization. PUS score thresholds: Colonization: 0; Mild: >0-2; Moderate: 2-5; Severe: ≥5.

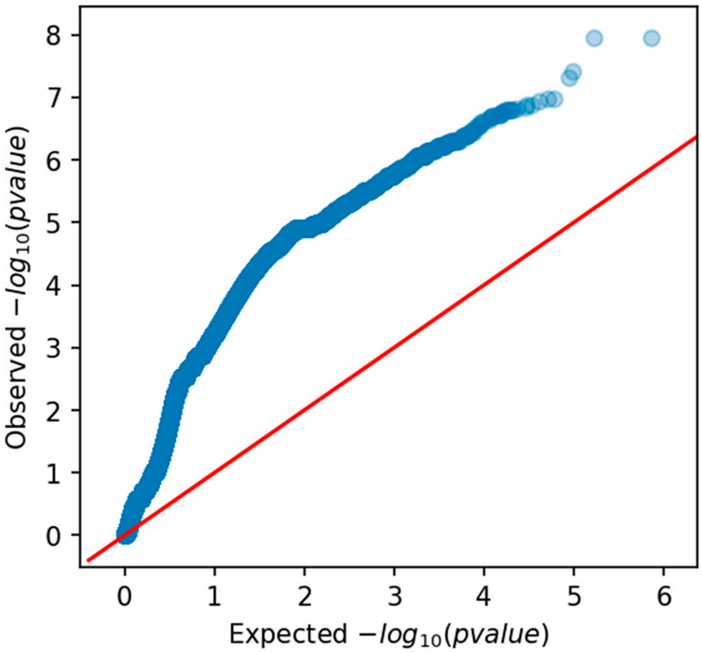

Figure S13. QQ-plot of unitigs associated with PUS score.

All unitigs identified in the association study were annotated with two reference genomes (NCTC 8325 and USA300) with the BWA alignment tool, resulting in 258,510 annotated unitigs (85% of the total 303,442). Of these, 50 were significantly associated with PUS score. All unitigs transformed adjusted p-values and effect sizes were plotted as a volcano plot (**Figure S14**).

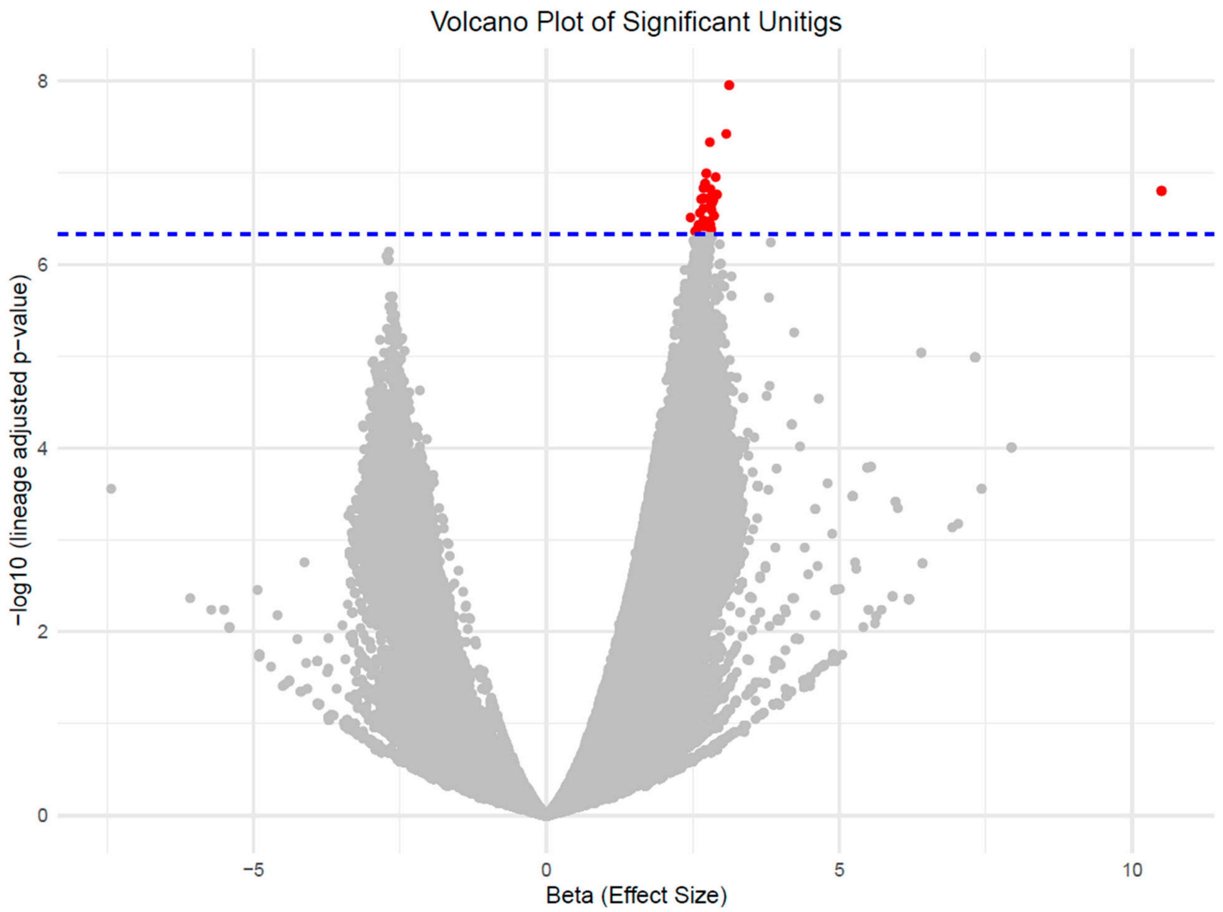

Figure S14. Volcano plot of all *Staphylococcus aureus* unitigs. Blue line at significant threshold (6.32; p=4.68E-07). Red dots relate to significantly associated unitigs to PUS score with positive effect sizes.

Supplemental Table S7. Annotation of Pyseer Identified *Staphylococcus aureus* Genes Associated with Increased PUS Score

Gene(s)	Description	Average Effect Size (PUS score)	Functional Annotation
ABD31075-ABD31076	Autolysin (LytO)-Holin	3.1	N-acetylmuramoyl-L-alanine amidase activity [GO:0008745]; cell wall organization [GO:0071555]; defense response to bacterium [GO:0042742]; killing of cells of another organism [GO:0031640]; peptidoglycan catabolic process [GO:0009253]; membrane [GO:0016020]
ABD30597-ABD30598	Autolysin (PH)-Holin	2.8	N-acetylmuramoyl-L-alanine amidase activity [GO:0008745]; cell wall organization [GO:0071555]; defense response to bacterium [GO:0042742]; killing of cells of another organism [GO:0031640]; peptidoglycan catabolic process [GO:0009253]; membrane [GO:0016020]
ABD30607	Lysostaphin	2.5	metallopeptidase activity [GO:0008237]
ABD31138	Hypothetical phage protein	2.2	Helix-xenobiotic response element family of transcriptional regulators
ABD30598	Holin	2.2	membrane [GO:0016020]
ABD31801	Surface protein G	2.1	extracellular region [GO:0005576]; single-species submerged biofilm formation [GO:0090609]
ABD31226	Lysostaphin	2.1	membrane [GO:0016020]; metallopeptidase activity [GO:0008237]
ABD31215	Peptidoglycan hydrolase	2.0	hydrolase activity [GO:0016787]
ABD30728	Penicillin-binding protein 3	2.0	plasma membrane [GO:0005886]; penicillin binding [GO:0008658]; peptidoglycan L,D-transpeptidase activity [GO:0071972]; cell wall organization [GO:0071555]
ABD30597	Autolysin (PH)	1.9	N-acetylmuramoyl-L-alanine amidase activity [GO:0008745]; cell wall organization [GO:0071555]; defense response to bacterium [GO:0042742]; killing of cells of another organism [GO:0031640]; peptidoglycan catabolic process [GO:0009253]
ABD30577	GTPase Der (EngA)	1.9	GTP binding [GO:0005525]; ribosome binding [GO:0043022]; ribosome biogenesis [GO:0042254]
ABD31272	Anti-repressor	1.8	
ABD31075	Autolysin (LytO)	1.8	N-acetylmuramoyl-L-alanine amidase activity [GO:0008745]; cell wall organization [GO:0071555]; defense response to bacterium [GO:0042742]; killing of cells of another organism [GO:0031640]; peptidoglycan catabolic process [GO:0009253]
ABD31221	Hypothetical phage protein	1.8	membrane [GO:0016020]
ABD31271	Anti-repressor	1.5	DNA binding [GO:0003677]

Abbreviation: GO: Gene ontology. Effect sizes are averages of unitig beta values which mapped within specified gene(s).

Supplemental Table S8. MLST, spa, SCCmec, SSTI and wound size

sample	SSTI	Wound Size (cm)	MLST	*spa* type	meca	SCC IVa	SCC IIb	SCC IVh
SAMN47787121	1	4	8	t008	1	0	0	0
SAMN47787185	1	2	8	t7477	1	1	0	0
SAMN47787125	1	2	8	t008	1	1	0	0
SAMN47787123	1	8	8	t008	1	1	0	0
SAMN47787115	1	3	8	t574	1	1	0	0
SAMN47787148	1	11	8	novel	1	1	0	0
SAMN47787192	1	3	8	t723	1	1	0	0
SAMN47787122	1	6	8	t008	1	1	0	0
SAMN47787161	1	2	8	t008	1	1	0	0
SAMN47787217	1	7	novel	t008	1	1	0	0
SAMN47787140	1	7	8	t008	1	1	0	0
SAMN47787183	1	3	novel	t622	1	1	0	0
SAMN47787128	1	10	novel	t2104	1	1	0	0
SAMN47787207	1	10	8	novel	1	1	0	0
SAMN47787147	1	2	8	t008	1	1	0	0
SAMN47787130	1	1	novel	novel	1	1	0	0
SAMN47787174	1	5	novel	t008	1	1	0	0
SAMN47787132	1	11	8	t008	1	1	0	0
SAMN47787218	1	6	8	t622	1	1	0	0
SAMN47787100	1	5	8	t622	1	1	0	0
SAMN47787216	1	4	8	t723	1	1	0	0
SAMN47787168	1	2	novel	t723	1	0	0	1
SAMN47787136	1	3	8	t723	1	1	0	0
SAMN47787156	1	6	8	t622	1	1	0	0
SAMN47787152	1	6	7776	t008	1	1	0	0
SAMN47787194	1	1	8	t304	1	1	0	0
SAMN47787113	1	2	8	t4407	1	1	0	0
SAMN47787124	1	7	8	t008	1	0	0	0
SAMN47787215	1	1	6370	t622	1	1	0	0
SAMN47787131	1	2	8	t622	1	1	0	0
SAMN47787204	1	5	novel	t14027	1	1	0	0
SAMN47787150	1	5	8	novel	1	1	0	0
SAMN47787199	1	3	8	t008	1	1	0	0
SAMN47787186	1	4	8	t008	1	1	0	0
SAMN47787108	1	6	novel	t008	1	1	0	0
SAMN47787155	1	3	8	t622	1	1	0	0
SAMN47787196	1	5	8	t008	1	1	0	0
SAMN47787129	1	9	8	t008	1	1	0	0
SAMN47787176	1	3	novel	t622	1	1	0	0
SAMN47787145	1	4	8	t2427	1	1	0	0

SAMN47787114	1	11	8	t024	1	1	0	0
SAMN47787172	1	7	8	novel	1	1	0	0
SAMN47787219	1	4	8	novel	1	1	0	0
SAMN47787104	1	6	8	t9121	1	1	0	0
SAMN47787167	1	2	8	t7477	1	1	0	0
SAMN47787191	1	4	8	t723	1	1	0	0
SAMN47787143	1	5	8	novel	1	1	0	0
SAMN47787197	1	2	8	t1578	1	1	0	0
SAMN47787159	1	9	8	t9325	1	1	0	0
SAMN47787214	1	6	novel	novel	1	1	0	0
SAMN47787133	1	17	novel	t723	1	1	0	0
SAMN47787206	1	2	novel	t17749	1	1	0	0
SAMN47787166	1	3	novel	t19002	1	1	0	0
SAMN47787202	1	9	8	t008	1	1	0	0
SAMN47787208	1	2	8	t008	1	1	0	0
SAMN47787107	1	6	8	t5649	1	1	0	0
SAMN47787103	1	14	8	t008	1	1	0	0
SAMN47787175	1	1	8	t622	1	1	0	0
SAMN47787224	1	3	8	t008	1	1	0	0
SAMN47787171	1	7	8	t622	1	1	0	0
SAMN47787119	1	1	8	t118	1	1	0	0
SAMN47787173	1	4	8	t622	1	1	0	0
SAMN47787201	1	12	8	t024	1	1	0	0
SAMN47787209	1	9	novel	t2743	1	1	0	0
SAMN47787149	1	4	8	t622	1	1	0	0
SAMN47787151	1	6	8	t1911	1	1	0	0
SAMN47787180	1	3	8	t723	1	1	0	0
SAMN47787162	1	7	8	t008	1	1	0	0
SAMN47787189	1	3	8	t622	1	1	0	0
SAMN47787213	1	4	8	t334	1	1	0	0
SAMN47787102	1	12	8	t008	1	1	0	0
SAMN47787141	1	14	8	t2229	1	1	0	0
SAMN47787165	1	10	8	t008	1	1	0	0
SAMN47787221	1	11	8	t622	1	1	0	0
SAMN47787184	1	4	novel	t334	1	1	0	0
SAMN47787225	1	5	8	t622	1	1	0	0
SAMN47787181	1	3	6370	t008	1	1	0	0
SAMN47787222	1	3	8	t008	1	1	0	0
SAMN47787187	1	5	8	t304	1	1	0	0
SAMN47787211	1	5	8	t008	1	1	0	0
SAMN47787120	1	7	8	t4407	1	1	0	0
SAMN47787142	1	2	8	t622	0	0	0	0
SAMN47787157	1	3	72	t4359	0	0	0	0

SAMN47787139	1	7	4236	t002	0	0	0	0
SAMN47787178	1	5	novel	novel	0	0	0	0
SAMN47787195	1	3	8	t024	0	0	0	0
SAMN47787116	1	10	8	t008	0	0	0	0
SAMN47787200	1	6	5	t002	0	0	0	0
SAMN47787153	1	4	45	t277	0	0	0	0
SAMN47787203	1	3	8	novel	0	0	0	0
SAMN47787126	1	4	188	t189	0	0	0	0
SAMN47787220	1	5	15	t094	0	0	0	0
SAMN47787101	1	7	8	t15459	0	0	0	0
SAMN47787144	1	8	8	t008	0	0	0	0
SAMN47787188	1	6	8	t008	0	0	0	0
SAMN47787193	1	3	8	t6238	0	0	0	0
SAMN47787117	1	3	8	t008	0	0	0	0
SAMN47787146	1	1	novel	t17749	0	0	0	0
SAMN47787138	1	6	novel	t008	0	0	0	0
SAMN47787169	1	6	45	t132	0	0	0	0
SAMN47787137	1	6	8	novel	0	0	0	0
SAMN47787106	1	5	8	t334	0	0	0	0
SAMN47787111	1	6	8	t059	0	0	0	0
SAMN47787112	1	9	8	t723	0	0	0	0
SAMN47787127	1	6	8	t4229	0	0	0	0
SAMN47787118	1	3	8	t1259	0	0	0	0
SAMN47787164	1	6	8	t622	0	0	0	0
SAMN47787158	1	6	59	t316	0	0	0	0
SAMN47787210	1	1	12	t160	0	0	0	0
SAMN47787154	1	4	121	t6465	0	0	0	0
SAMN47787110	1	15	8	t9829	0	0	0	0
SAMN47787160	1	5	novel	t160	0	0	0	0
SAMN47787223	1	1	20	t1987	0	0	0	0
SAMN47787179	1	2	188	t189	0	0	0	0
SAMN47787134	1	5	8	t008	0	0	0	0
SAMN47787212	1	1	novel	t334	0	0	0	0
SAMN47787198	1	6	4236	t111	0	0	0	0
SAMN47787163	1	12	novel	t111	0	0	0	0
SAMN47787170	1	2	8	t723	0	0	0	0
SAMN47787177	1	10	8	t622	0	0	0	0
SAMN47787109	1	6	8	t008	0	0	0	0
SAMN47787205	1	3	novel	t008	0	0	0	0
SAMN47787182	1	2	15	t084	0	0	0	0
SAMN47787105	1	3	8	t622	0	0	0	0
SAMN47787190	1	7	5	t062	0	0	0	0
SAMN47787135	1	4	59	t216	0	0	0	0

SAMN47787237	0	0	8	t351	1	1	0	0
SAMN47787245	0	0	8	t622	1	1	0	0
SAMN47787255	0	0	8	t622	1	1	0	0
SAMN47787226	0	0	novel	novel	1	0	1	0
SAMN47787246	0	0	5	t111	0	0	0	0
SAMN47787240	0	0	5	t688	0	0	0	0
SAMN47787243	0	0	30	t129	0	0	0	0
SAMN47787251	0	0	5	novel	0	0	0	0
SAMN47787248	0	0	25	t287	0	0	0	0
SAMN47787242	0	0	5	t111	0	0	0	0
SAMN47787254	0	0	30	t2306	0	0	0	0
SAMN47787231	0	0	188	t189	0	0	0	0
SAMN47787232	0	0	15	t1877	0	0	0	0
SAMN47787250	0	0	5	t111	0	0	0	0
SAMN47787253	0	0	45	t918	0	0	0	0
SAMN47787238	0	0	6	t5271	0	0	0	0
SAMN47787256	0	0	30	novel	0	0	0	0
SAMN47787235	0	0	1159	t091	0	0	0	0
SAMN47787241	0	0	45	t147	0	0	0	0
SAMN47787236	0	0	45	novel	0	0	0	0
SAMN47787249	0	0	5	novel	0	0	0	0
SAMN47787244	0	0	30	t275	0	0	0	0
SAMN47787234	0	0	188	t189	0	0	0	0
SAMN47787247	0	0	45	novel	0	0	0	0
SAMN47787230	0	0	novel	t267	0	0	0	0
SAMN47787228	0	0	30	t338	0	0	0	0
SAMN47787239	0	0	30	t338	0	0	0	0
SAMN47787252	0	0	30	t871	0	0	0	0
SAMN47787227	0	0	45	t282	0	0	0	0
SAMN47787229	0	0	novel	t008	0	0	0	0
SAMN47787233	0	0	5	t1303	0	0	0	0

Supplemental Text S1. Metagenomic results

From the nasal metagenomic assembly, we recovered a set of genomic bins with varying completeness and contamination; applying conventional "high-quality" criteria (e.g., completeness ≥90% and low contamination) yields seven well-resolved bins dominated by *Enterococcus* (bin 7), *Micrococcus* (bin 2), and multiple *Staphylococcus* species (bins 3, 4, 5, 8, 9), with completeness largely above 84% (several exceeding 95%) and minimal contamination (**Supplemental Data 3**). These taxa reflect common nasal commensals and opportunistic pathogens, with *Staphylococcus aureus* and related species, Enterococcus faecalis (or close relatives), and *Micrococcus* contributing the bulk of the high-quality genomic content. The integrated summary table combines taxonomic/quality metadata with functional annotations from core metabolism (GO), accessory gene families (COG, EC), and virulence factor profiling (VFDB), enabling a unified view of retained essential biology alongside potential pathogenicity signatures. All high-quality bins uniformly retain the defined set of 94 core GO biological process terms, which have been grouped into higher-order themes to facilitate interpretation. Major retained core functions include amino acid biosynthesis, genome maintenance and repair, central carbon and energy metabolism, nucleotide metabolism, transcription and RNA processing, translation and protein maturation, transport mechanisms, stress response/homeostasis, and cell envelope/division processes. The presence of these core themes across taxa—even in the face of some variation in completeness—underscores a conserved backbone of essential metabolism required for persistence in the nasal niche. A large fraction of GO terms remain "unassigned/other," reflecting either process granularity beyond the grouped themes or unresolved annotations, but the key biosynthetic and maintenance pathways are robustly preserved, indicating metabolic self-sufficiency rather than extreme dependency.

Beyond the core, the bins differ markedly in accessory capacity. *Enterococcus* (bin 7) exhibits the richest complement of accessory functions, with over a thousand COGs detected and a correspondingly expansive set of EC-numbered enzymatic activities, suggesting substantial metabolic flexibility and potentially niche-adaptive specialization. *Micrococcus* also maintains a large accessory repertoire, albeit somewhat smaller, while individual *Staphylococcus* bins vary—some showing moderately reduced counts consistent with either assembly fragmentation or narrower functional breadth. The detected EC numbers span central and peripheral metabolic reactions, indicating active participation in diverse biochemical conversions, including those involved in cofactor metabolism, energy production, and substrate interconversion. The breadth of COG representation further implies that these organisms retain both housekeeping accessory functions and potential adaptive gene sets that could mediate environmental sensing, transport modulation, and interactions with host-derived substrates.

Virulence profiling reveals distinct patterns across taxa. *Enterococcus* (bin 7) harbors a rich set of virulence-associated genes, including components of the cytolysin system (e.g., *cylA*, *cylB*, *cylI*, *cylM*, and regulator *cylR1*), adhesion and aggregation machinery (e.g., *efaA*, aggregation substance EF0149), and immune modulation factors (capsule-related enzymes such as *cpsA*/*cdsA*). These factors appear in combinations that could promote host adherence, biofilm formation, immune evasion, and cytotoxicity—painting a picture of a flexible opportunist capable of transitioning toward pathogenic interactions under favorable conditions. *Staphylococcus* bins include well-known virulence determinants such as hemolysins (gamma-hemolysin components *hlgB*, *hlgC*), biofilm-related genes (e.g., *icaA*), and surface adhesins, consistent with their roles in colonization and immune interaction. Some of these virulence factors are shared among bins (reflecting either close phylogenetic relatedness or horizontal inheritance), while others are

unique, highlighting bin-specific virulence repertoires that could influence differential clinical relevance or competitive dynamics. *Micrococcus* in this dataset shows relatively sparse classical virulence factor annotation, aligning with its common commensal status, though its accessory and core metabolic retention suggest ecological resilience.

Dissecting by GO theme, the strong retention of amino acid biosynthesis pathways suggests that these organisms are largely prototrophic, reducing reliance on external amino acid pools and supporting stable colonization. Genome maintenance and repair genes are abundant, which may help persistence under oxidative and host-imposed stress in the nasal environment. Transport-related functions complement metabolic capacity, enabling uptake and efflux of diverse molecules, possibly including host-derived nutrients or antimicrobial compounds. Stress response/homeostasis genes bolster survival in the fluctuating mucosal habitat. From the COG perspective, the presence of diverse families associated with transcription regulation, signal transduction, and membrane processes speaks to adaptive responsiveness. EC-level annotations further refine this picture: *Enterococcus* and *Staphylococcus* display enzymatic activities consistent with robust central metabolism (e.g., dehydrogenases, kinases) but also possess enzymes likely involved in detoxification and cofactor turnover, which could modulate host-microbe and microbe-microbe interactions.

One particularly interesting finding is the juxtaposition of extensive core and accessory functionality with taxon-specific virulence repertoires: *Enterococcus* combines metabolic breadth with potent adhesion/toxin systems, positioning it as a candidate for opportunistic transition under immune compromise. *Staphylococcus* species show variable accessory gene loads yet retain key colonization and toxin modules, highlighting divergent evolutionary strategies to thrive in the same niche.

Overall, the integrated virulence + core function summary reveals a nasal microbiome subset where metabolic self-sufficiency and adaptability coexist with lineage-specific virulence potential. This duality—core metabolic robustness paired with selective virulence determinants—suggests that these organisms occupy a stable commensal baseline but carry the genetic toolkit for pathogenesis when ecological or host conditions shift. The fine-grained annotation across GO, COG, EC, and VFDB axes provides a rich framework for prioritizing bins or taxa for further experimental validation, surveillance, or clinical correlation.
